# Intrathecal morphine vs. Ultrasound-guided bilateral posterior quadratus lumborum block in caesarean delivery

**DOI:** 10.1186/s44158-025-00235-0

**Published:** 2025-03-06

**Authors:** Burhan Dost, Hilal Hanife Kandemir, Kubra Tabur, Sule Nur Karakurt, Beliz Yayla, Canan Asar Sahin, Cengiz Kaya

**Affiliations:** Department of Anesthesiology and Reanimation, School of Medicine, Ondokuz Mayis University, Samsun, Türkiye

**Keywords:** Cesarean Section, Nerve block, Opioid, Pain management, Postoperative, Ultrasonography

## Abstract

**Background:**

Effective postoperative pain management is crucial in caesarean delivery (CD) to enhance recovery, minimize opioid use, and improve maternal outcomes. Intrathecal morphine (ITM) is widely used but can cause side effects, such as pruritus and nausea. Posterior quadratus lumborum block (QLB) has emerged as a potential alternative for postoperative analgesia. This study compared the analgesic efficacy and side-effect profiles of ITM and posterior QLB in patients with CD.

**Methods:**

This prospective observational study included parturients who underwent elective CD under spinal anesthesia. Participants were allocated to receive either ITM (100 µg) or bilateral posterior QLB with 0.25% bupivacaine (25 mL per side). The primary outcome was cumulative intravenous morphine consumption 24 h post-surgery. The secondary outcomes included NRS pain scores at rest and during activity at 0, 3, 6, 12, and 24 h, the time to first opioid request, the number of patients requiring rescue analgesia, nausea and vomiting scores, pruritus scores, and scores on the Obstetric Quality of Recovery Scale (ObsQoR-11 T) at 24 h and 48 h postoperatively.

**Results:**

Sixty patients were included in the analysis, with 30 patients in each group. The primary outcome, 24-h cumulative intravenous morphine consumption, was comparable between the ITM and posterior QLB groups (6 [10] mg vs. 8.2 [7.1] mg, *p* = 0.134). The secondary outcomes, including NRS pain scores at rest and during activity, time to first opioid request, number of patients requiring rescue analgesia (1 vs. 0; *p* = 0.313), nausea and vomiting scores, pruritus scores (0 [1] vs. 0 [0]; *p* = 0.234), and ObsQoR-11 T scores at 24 h (95.5 [14] vs. 87.5 [16]; *p* = 0.49) and 48 h (102 [13] vs. 97 [18]; *p* = 0.203), were not significantly different between the groups.

**Conclusion:**

Both ITM and posterior QLB provide effective postoperative analgesia in patients with CD, with comparable analgesic outcomes and side-effect profiles. ITM remains a practical choice because of its ease of administration, whereas subsequent QLB serves as a viable alternative for patients intolerant to neuraxial opioids.

## Introduction

Moderate to severe postoperative pain is commonly related to caesarean delivery (CD), which can seriously impair daily activities, healing, and interactions between the mother and newborn [1]. Concerns regarding the potential side effects of analgesic treatments on both mothers and neonates often lead to suboptimal pain management. This insufficiency in pain control is not merely a discomfort issue but also poses substantial clinical risks, including impaired physical recovery, delayed return to normal activities, and potential negative psychological outcomes, such as increased anxiety and postpartum depression. Moreover, inadequate pain management may contribute to the development of chronic postoperative pain syndromes, increased thromboembolic events due to reduced mobility, and hyperalgesia, further complicating the postpartum period [2, 3]. Consequently, effective and safe analgesic strategies that allow mothers to care for their newborns without adverse effects are critically important.

Various multimodal analgesic techniques have been employed to optimize pain relief following CD with the aim of minimizing opioid consumption while providing effective analgesia. Neuraxial methods, local anesthetic infiltration, and fascial plane blocks, such as quadratus lumborum block (QLB), erector spinae plane (ESP) block, and transversus abdominis plane (TAP) block, have demonstrated varying degrees of efficacy [4, 5]. Current guidelines from the procedure-specific postoperative pain management (PROSPECT) group recommend local anesthetic wound infiltration, continuous wound infusion, and/or fascial plane blocks such as TAP, ESP, and QLB when intrathecal morphine (ITM) is not used [6]. However, the relative effectiveness of these approaches, particularly between the ITM and the posterior QLB, remains an area of active investigation. This study aimed to compare the analgesic efficacy and side-effect profiles of ITM and posterior QLB in managing acute postoperative pain following CD.

## Method

This prospective, single-center, observational study was conducted at Ondokuz Mayis University between July and November 2024, following approval from the Ondokuz Mayis University Faculty of Medicine Ethics Committee (approval number: 2024/237). The study was registered at https://www.clinicaltrials.gov/ prior to the enrollment of the first patient (Registration No: NCT06481462, on June 25th 2024). All the procedures were performed in accordance with the principles of the Declaration of Helsinki. The Strengthening the Reporting of Observational Studies in Epidemiology (STROBE) guidelines [7] were also followed in the preparation of this study.

After providing written informed consent, pregnant women who met the following inclusion criteria were enrolled: gestational age of at least 37 weeks, age between 18 and 45 years, American Society of Anesthesiologists (ASA) physical status score II, and planned for elective CD under spinal anesthesia. The exclusion criteria included patients with an ASA score of III or IV, those scheduled for CD under general anesthesia, those requiring conversion to general anesthesia after failed spinal anesthesia, those with contraindications to spinal anesthesia and regional anesthesia techniques, those with a body mass index greater than 35 kg/m^2^, those with a history of opioid use disorder or opioid use for more than four weeks, those unable to assess pain scores, those with a gestational age of less than 37 weeks, those with a history of allergy to local anesthetics or systemic opioids, and those who declined to participate in the study.

In this prospective observational study, patients who met the inclusion criteria were consecutively enrolled until the desired sample size was achieved. Allocation to either the ITM or posterior QLB group was determined by the clinical judgment of the anesthesiologist and patient preference. For example, if a patient declined ITM due to previous experience or other reasons, they were offered the option of a posterior QLB block. Group assignments were concealed from the research team to maintain the integrity of the study's results.

### Anesthesiological management

In this study, institutional perioperative care protocols were implemented to ensure patient safety and optimal recovery. Antibiotic prophylaxis was administered according to the institutional guidelines to minimize the risk of surgical site infections. Vital parameters (heart rate, blood pressure, oxygen saturation, and temperature) were continuously monitored both intraoperatively and postoperatively. Fluid management was individualized based on the patient’s needs and hemodynamic status. For postoperative nausea and vomiting (PONV) prophylaxis, all patients received 4 mg IV dexamethasone following delivery, followed by 0.1 mg/kg IV ondansetron before skin closure. To ensure adequate uterine contraction and minimize bleeding, 100 µg carbetocin was administered intraoperatively per the obstetric protocol.

## Procedures

### Spinal anesthesia procedure

In both groups, the patients were seated after standard ASA monitoring (electrocardiography, noninvasive arterial blood pressure, and peripheral oxygen saturation). Under sterile conditions, spinal anesthesia was administered via a 26G spinal needle inserted at the L4–L5 intervertebral space, which reached the subarachnoid space. A subarachnoid block was achieved by injecting 12.5 mg of 0.5% hyperbaric bupivacaine and 20 µg fentanyl. In the ITM group, 100 µg of morphine was added to the solution. The sensory block level of spinal anesthesia was confirmed to reach T6 using the pinprick test before initiating CD. Additionally, the Hollmén scale was used to evaluate the depth of the sensory block, whereas motor block assessment was performed using the Bromage scale to ensure a comprehensive evaluation of the anesthetic effect.

### Ultrasound-guided posterior QLB block

**Fig. 1:**
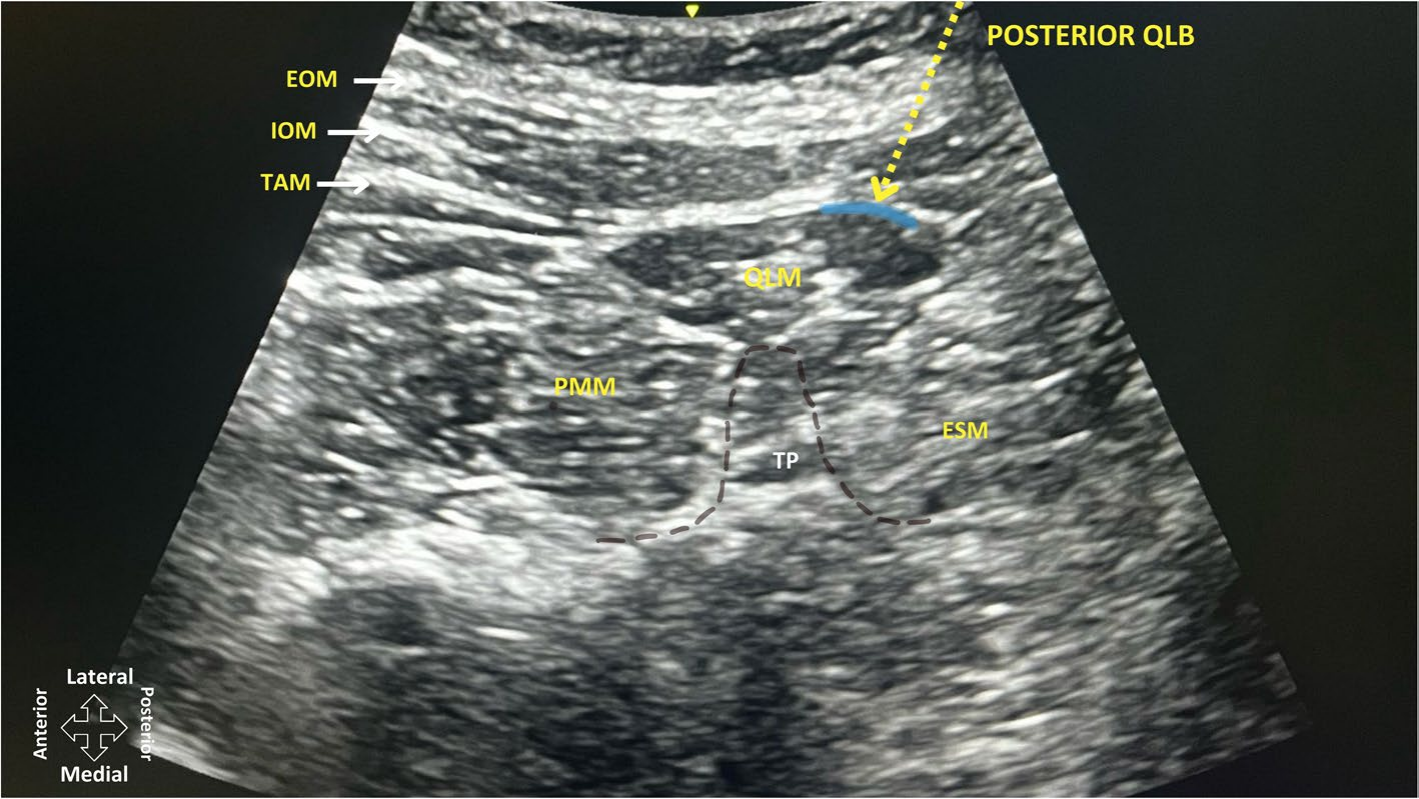
Flow diagram of the participants. Abbreviations: QLB, quadratus lumborum block; ITM, intrathecal morphine

At the end of the CD, the patients were positioned laterally and sterile conditions were ensured. A convex ultrasound transducer (2–5 MHz, GE LOGIQ V1 Ultrasound System, China) was placed transversely in the midline between the iliac crest and subcostal region. The abdominal muscles, latissimus dorsi muscle, erector spinae muscle, psoas muscle, transverse process of the fourth lumbar vertebra, and vertebral body were identified sonographically (Fig. 1). 25 mL of 0.25% bupivacaine was administered into the fascial plane between the quadratus lumborum muscle and the latissimus dorsi muscle. The patient was then repositioned to the opposite side, and the procedure was repeated via the same technique, method, and volume of local anesthetic. All blocks were performed by experienced anesthesiologists who had previously completed at least 20 successful posterior QLBs without complications. Data collection was carried out by a resident who was blinded to the patients' group assignments and was not involved in their clinical care during the study.

### Pain management

During the preoperative visit, patients were educated about patient-controlled analgesia (PCA) and the NRS for pain assessment. The NRS is a 10 cm visual scale, with "no pain" at one end and "the most severe pain imaginable" at the other. Patients were instructed to rate their pain intensity via this scale.

Intraoperatively, patients received 20 mg of tenoxicam following induction, 1 g of intravenous paracetamol before the end of surgery, and 1 g of intravenous paracetamol every 8 h postoperatively. The PCA device (BodyGuard 575 Pain Manager, BD) was programmed to deliver morphine at a dose of 20 µg/kg with a 10-min lockout interval and a 4-h limit to 80% of the maximum achievable dose, with no continuous infusion.

All patients were provided with a PCA device in the recovery unit. For breakthrough pain (NRS ≥ 4 despite PCA usage), rescue analgesia was administered as a 30-min infusion of 100 mg intravenous tramadol, with a maximum limit of 300 mg/day. Pain scores at rest and during activity (e.g., coughing or deep inspiration) were recorded at 3, 6, 12, and 24 h postoperatively in the PACU. Additionally, the number of patients requiring rescue analgesia and the time at which PCA analgesia was first requested were documented.

### Outcomes

The primary outcome was total intravenous morphine consumption within the first 24 h after surgery. The secondary outcomes included NRS pain scores at rest and during activity at 0, 3, 6, 12, and 24 h; the number of patients requiring rescue analgesia; nausea and vomiting scores; pruritis scores; and scores on the Obstetric Quality of Recovery Scale (ObsQoR-11 T) at 24 h and 48 h postoperatively. Additional data collected included block-related complications (such as hematoma, infection, pneumothorax, and local anesthetic toxicity).

### PONV

PONV was evaluated via a verbal descriptive scale (0 = none; 1 = mild nausea; 2 = moderate nausea; 3 = vomiting once; 4 = vomiting more than once). Patients with a score ≥ 3, 4 mg IV ondansetron were administered.

### Pruritus assessment

Pruritus was assessed using a four-point scale to determine the severity of itching (0 = no pruritus; 1 = mild, represented a sensation of itching without scratching, only a rubbing sensation; 2 = moderate, denoted a sensation of itching with active scratching; and 3 = severe, reflected a sensation of itching with scratching that necessitated treatment) [8]. When the score is > 2, patients are administered IV diphenhydramine 25–50 mg, and if it persists, IV prednisolone 0.5 mg/kg is administered.

### Quality of postoperative recovery and patient satisfaction

Patient satisfaction and the quality of postoperative recovery in all patients from both groups were assessed via the Turkish version of the Obstetric Quality of Recovery Scale (ObsQoR-11 T). This scale comprises 11 items designed to evaluate key aspects of recovery, including physical comfort, emotional well-being, pain management, ability to mobilize, energy levels, breastfeeding satisfaction, and overall satisfaction with care. The scale aims to provide a comprehensive assessment of the patient's recovery experience and outcomes specific to the obstetric population [9].

The sample size was calculated by G*Power statistical software (version 3.1.9.6; Universität Kiel, Germany). On the basis of the mean and standard deviation (SD) values for 24-h morphine consumption reported in previous studies [10, 11]— 11 (11.01) in the posterior QLB group and 2.7 (4.8) in the ITM group—the effect size was calculated as 0.97. Assuming a Type I error (α) of 5% and a study power of 95%, it was estimated that 29 patients would be required in each group. To allow for potential data loss, the sample size was increased to 30 patients per group.

### Statistical analysis

Statistical analysis was performed using SPSS version 28.0 (IBM Corporation). The Kolmogorov–Smirnov test was employed to evaluate the normality of variable distributions. Continuous data are presented as the means ± standard deviations and medians (interquartile ranges [IQRs]), whereas categorical data are reported as frequencies (n) and percentages (%). For comparisons between the two groups, categorical variables were analyzed using the chi-square test or Fisher’s exact test, as appropriate. Continuous variables were evaluated based on their distribution. Normally distributed variables were compared using the independent samples t-test, whereas non-normally distributed data were analyzed using the Mann–Whitney U test. Statistical significance was defined as *p* < 0.05.

## Results

**Fig. 2:**
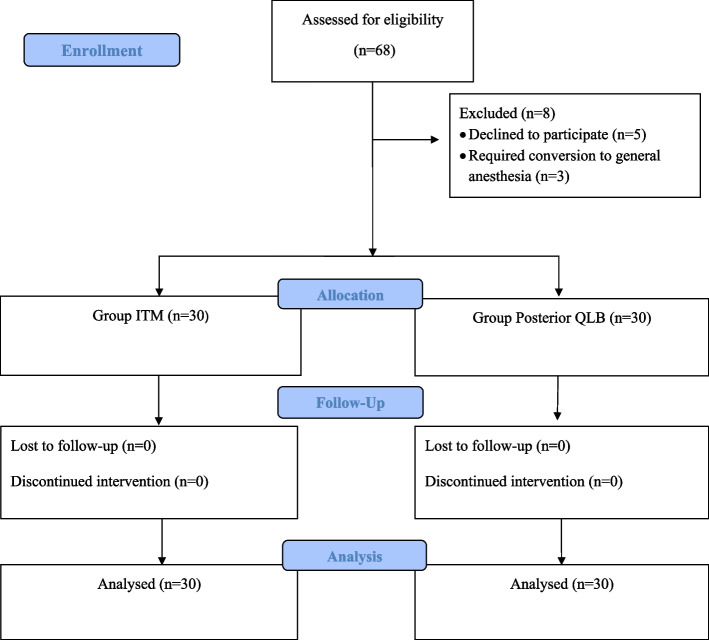
Flow Diagram of the Study Population

A total of 68 patients were enrolled in the study. Eight patients were excluded: five declined to participate and three were excluded because of conversion to general anesthesia. Finally, data from 60 patients were included in the analysis, with 30 and 30 patients in the ITM and posterior QLB groups, respectively (Fig. 2). The demographic characteristics of the two groups are presented in Table 1. Cumulative intravenous morphine consumption within the first 24 h was slightly greater in the posterior QLB group than in the ITM group (8.2 [7.1] mg vs. 6 [10] mg), but this difference was not statistically significant (*p* = 0.134) (Table 1). The median NRS pain scores at rest and during activity were comparable at all time points (0, 3, 6, 12, and 24 h) between groups (*p* > 0.05) (Table 2).

**Table 1:** Demographic and clinical characteristics

	Group ITM (*n* = 30)	Group QLB (*n* = 30)	**P**
Age (years)	30 ± 3.86	27.5 ± 5.14	
BMI (kg/m^2^)	29.57 ± 3.92	28.27 ± 5.23	
Surgery time (min)	65 (25)	60 (55)	
Comorbidities (n, %)			0.849
None	22 (73)	23 (76)	
Cardiovascular system disorders^a^	0	0	
Endocrine system disorders^b^	4 (13)	5 (17)	
Respiratory system disorders^c^	2 (7)	1 (3)	
Other	2 (7)	1 (3)	
Primipara (n, %)	16 (53)	19 (63)	0.432
Morphine consumption (mg)	6 (10)	8.2 (7.1)	0.134
Time to first morphine request (min)	160 (300)	135 (90)	0.177
Patients given rescue analgesics in the first 24 h (n, %)	1 (3)	0 (0)	0.313
Pruritis score	0 (1)	0 (0)	0.234
ObsQoR-11 T Scores 24th h	95.5 (14)	87.5 (16)	0.490
ObsQoR-11 T Scores 48th h	102 (13)	97 (18)	0.203

Continuous variables are presented as mean ± SD or median (IQRs), and categorical variables are presented as counts (%)

^a^Hypertension, coronary artery disease

^b^Type 2 diabetes, goiter

^c^Allergic asthma *ITM*, Intrathecal morphine, *QLB* Quadratus Lumborum Block, *BMI* Body mass index, *ObsQoR-11 T* Obstetric quality of recovery scale

**Table 2:** Comparison of NRS scores at rest and during activity in the first 24 h

	Group ITM (*n* = 30)	Group QLB (*n* = 30)	**P**
NRS_Rest_			
0th h	2.56 (1.35)	2.56 (1.03)	0.633
3rd h	2.90 (1.14)	2.56 (1.03)	0.419
6th h	3.17 (0.96)	3.30 (1.36)	0.082
12th h	2.73 (1.24)	3.30 (1.36)	0.225
24th h	2.30 (1.34)	2.30 (1.34)	0.781
NRS_Activity_			
0th h	2.37 (1.32)	0.44 (0.73)	0.504
3rd h	3.73 (1.55)	3.97 (1.32)	0.664
6th h	4.20 (1.24)	4.30 (1.54)	0.227
12th h	3.60 (1.40)	3.60 (1.40)	0.161
24th h	3.17 (1.71)	3.20 (1.46)	0.799

Continuous variables are presented as medians (IQRs)

*ITM* Intrathecal morphine, *QLB* Quadratus lumborum block

The incidence of PONV requiring antiemetics was slightly higher in the ITM group (5 vs. 1), but this difference was not statistically significant (*p* = 0.85). The PONV scores did not differ significantly between the groups at any time point (*p* > 0.05) (Table 3). Pruritus was more common in the ITM group (eight vs. four patients), but the difference was not statistically significant (*p* = 0.333). The pruritus scores were also comparable between the groups (*P* = 0.234) (Table 1). Only one patient in the ITM group required rescue analgesia, whereas none of the patients in the QLB group required rescue analgesia (*p* = 0.313) (Table 1). The ObsQoR-11 T scores for recovery and patient satisfaction were similar at 24 and 48 h in both groups (*p* > 0.05) (Table 1). No block-related complications were observed in this study.

**Table 3:** Comparison of PONV scores among groups

	Group ITM (*n* = 30)	Group QLB (*n* = 30)	**P**
0th h	0.00 (0.00)	0.00 (0.00)	1.0
3rd h	0.00 (0.00)	0.00 (0.00)	0.91
6th h	0.00 (0.00)	0.04 (0.32)	0.154
12th h	0.07 (0.20)	0.04 (0.32)	0.289
24th h	0.00 (0.00)	0.07 (0.20)	0.557

Continuous variables are presented as medians (IQRs)

*ITM* Intrathecal morphine, *QLB* Quadratus lumborum block, *PONV* Postoperative nausea and vomiting

## Discussion

In our study comparing the analgesic efficacy of ITM and posterior QLB in patients with CD, we observed no significant difference in the primary outcome of 24-h cumulative morphine consumption between the two groups. Furthermore, secondary outcomes, including postoperative pain scores, time to first opioid request, nausea and vomiting scores, incidence of pruritus, and ObsQoR-11 T scores, were similar in both groups.

Literature on the efficacy of various types of QLBs in patients with CD continues to evolve. Although our previous study [4] and a recent network meta-analysis [12] suggested that anterior QLB may be superior, the technical difficulty and positional requirements for performing anterior QLB, even under ultrasound guidance, limit its routine use in our clinical practice. Consequently, in cases where neuraxial long-acting opioids are contraindicated or not feasible, posterior QLB is preferred, as recommended by the PROSPECT guidelines [6]. Consistent with our findings, Giral et al. [13] demonstrated in a randomized controlled trial that ITM and posterior QLB resulted in comparable 24-h cumulative intravenous morphine consumption. However, their secondary outcomes indicated that the QLB group had advantages over the ITM group in terms of time to first PCA morphine requirement, ObsQoR-11 scores, and pruritus incidence. Despite using a similar analgesia protocol, our study found that both techniques yielded comparable results in secondary outcomes as well. Although the literature suggests that a minimal clinically important difference (MCID) requires at least a 10 mg difference in morphine consumption or a 2-point difference in NRS/VAS scores [14], no such difference was observed in our study. Both groups failed to meet MCID thresholds. Therefore, the analgesic efficacies of the two techniques were not only statistically but also clinically comparable.

There were no significant differences between the groups in terms of PONV or pruritus scores, indicating a comparable side effect profile for both methods. Although ITM is typically associated with opioid-related adverse effects [15], these side effects were not significantly different between the groups in our study. This may be attributed to the use of a low dose of morphine in our protocol. A recent meta-analysis revealed that higher doses of intrathecal morphine (> 100 µg), while increasing the duration of analgesia, are more frequently associated with side effects [16]. Additionally, while posterior QLB requires ultrasound guidance, specialized needles, practitioner expertise, and an additional puncture, ITM is significantly simpler and quicker to administer, making it a more feasible option for postoperative pain management in patients with CD. This, in turn, could enhance patient comfort and satisfaction, underscoring the practicality of the ITM in this clinical context.

Postoperative pain management during obstetric anesthesia affects not only maternal comfort but also neonatal clinical outcomes. While our study primarily evaluated the effectiveness of maternal analgesia, it is important to consider that postoperative pain control may influence factors such as breastfeeding success and mother-infant bonding [17]. Additionally, systemic opioid use can lead to neonatal sedation and feeding difficulties, highlighting the potential advantages of regional anesthesia techniques in minimizing these risks.

Our study had several limitations. The single-center nature of our study limited the generalizability of our findings. Additionally, we were unable to perform sensory analysis following the block because of the residual effects of spinal anesthesia at the time of block application. This study's observational design, in which group allocation was based on clinical judgment and patient preference rather than randomization, may have introduced selection bias. In addition, prior knowledge of the chosen analgesic technique could have influenced pain perception and modulation, potentially affecting the subjective assessment of pain outcomes. However, this study design is valuable in reflecting real clinical practice, where patient and anesthesiologist preferences often guide the choice of regional anesthesia techniques. Furthermore, we did not evaluate long-term outcomes such as chronic pain or patient satisfaction beyond 24 h, which could provide a more comprehensive understanding of the two methods.

## Conclusion

Our study demonstrated that both ITM and posterior QLB provide effective postoperative analgesia in patients with CD, with no significant differences in morphine consumption or secondary outcomes, such as pain scores, nausea, vomiting, pruritus, or functional recovery. ITM remains a simpler and more practical option for postoperative pain management because of its ease of administration, proven efficacy with a low dose of opioids, and minimal side effects. However, posterior QLB may be a valuable option for patients who cannot receive neuraxial opioids or are particularly sensitive to side effects.

## Data Availability

The datasets used and/or analysed during the current study available from the corresponding author on reasonable request.
